# Radiopharmaceutical Formulation and Preclinical Testing of ^68^Ga-Labeled DOTA-MGS5 for the Regulatory Approval of a First Exploratory Clinical Trial

**DOI:** 10.3390/ph14060575

**Published:** 2021-06-16

**Authors:** Anton A. Hörmann, Maximilian Klingler, Christine Rangger, Christian Mair, Clemens Decristoforo, Christian Uprimny, Irene J. Virgolini, Elisabeth von Guggenberg

**Affiliations:** Department of Nuclear Medicine, Medical University of Innsbruck, 6020 Innsbruck, Austria; anton.hoermann@i-med.ac.at (A.A.H.); klinglermaximilian@gmail.com (M.K.); christine.rangger@tirol-kliniken.at (C.R.); c.mair@tirol-kliniken.at (C.M.); clemens.decristoforo@tirol-kliniken.at (C.D.); christian.uprimny@tirol-kliniken.at (C.U.); irene.virgolini@tirol-kliniken.at (I.J.V.)

**Keywords:** cholecystokinin-2 receptor, minigastrin, molecular imaging, radiometals, clinical trial, automated synthesis

## Abstract

The new minigastrin analog DOTA-MGS5 is a promising new candidate for targeting cholecystokinin-2 receptor (CCK2R)-expressing tumors. To enable the clinical translation of PET/CT imaging using ^68^Ga-labeled DOTA-MGS5, different quality and safety aspects need to be considered to comply with the regulatory framework for clinical trial application. The preparation of the radiopharmaceutical was established using a cassette-based automated synthesis unit. Product specifications, including analytical procedures and acceptance criteria, were adopted from Ph. Eur. monographs for other ^68^Ga-labeled radiopharmaceuticals. Non-clinical studies included receptor affinity and cell uptake studies using two different CCK2R-expressing cell lines, as well as pharmacokinetic biodistribution studies in BALB/c mice for dosimetry calculations and toxicological studies in Wistar rats. The produced masterbatches fulfilled the defined acceptance criteria. DOTA-MGS5, with confirmed affinity to the CCK2R, showed a high specific cell uptake and no interaction with other receptors in vitro when radiolabeled with gallium-68. Favorable in vivo properties were observed in biodistribution and dosimetry studies. An effective dose of ~0.01 mSv/MBq was estimated for humans utilizing OLINDA/EXM software. A maximum peptide dose of 50 µg was established for the initial clinical dose based on the toxicity study in rats. The standardized production of [^68^Ga]Ga-DOTA-MGS5 using an automated synthesis module and the performed non-clinical safety studies support a first exploratory clinical trial with this new PET imaging agent.

## 1. Introduction

In recent years, the use of ^68^Ga-labeled radiopharmaceuticals for positron emission tomography (PET) has become more prominent in clinical routine. This was fostered by the increased availability of gallium-68 from commercial ^68^Ge/^68^Ga generators. PET imaging based on ^68^Ga-labeled peptide analogs offers higher sensitivity and specificity compared with conventional gamma scintigraphy, allowing for enhanced diagnostic quality of malignant lesions [1,2,3]. In general, the preparation of ^68^Ga-labeled radiopharmaceuticals is accomplished by in-house production under standardized conditions to ensure consistent quality of the product. In addition to the radiometal complex formation, the process steps include purification of gallium-68 eluted from the ^68^Ge/^68^Ga-generator and formulation of the final injectable solution by solid-phase extraction. Automation of synthesis is usually applied due to the complexity of the whole process and the high radiation exposure during handling of gallium-68, a positron emitter with maximum energy of 1.92 MeV and half-life of 68 min [4]. The use of a cassette-based automated synthesis unit allows for efficient and rapid preparation of ^68^Ga-labeled radiopharmaceuticals with adequate radiation protection measures and in compliance with regulatory demands [5].

The clinical translation of highly innovative radiopharmaceuticals has to follow the regulatory framework for clinical trials in the European Union [6]. Different quality and safety aspects need to be considered before first-in-human application. In this respect, the European Pharmacopoeia (Ph. Eur.) provides a legal and scientific basis in defining quality standards [7]. The ICH guideline M3(R2) on non-clinical safety studies for the conduct of human clinical trials and marketing authorization for pharmaceuticals gives specific advice on the conduction of toxicity studies [8]. For PET imaging agents, the recommendations given for a single-dose microdose approach are applicable in support of a first exploratory clinical trial. The EANM guideline for the preparation of an Investigational Medicinal Product Dossier (IMPD) covers and discusses different challenging tasks in the preparation of quality documentation for a radiopharmaceutical drug [9]. In the section on the chemical and pharmaceutical quality, all documentation related to the drug substance (active pharmaceutical ingredient) and on the investigational medicinal product (IMP) under test needs to be provided. This includes detailed information on the chemical precursor for the radiopharmaceutical preparation and of the final radiopharmaceutical preparation. The second part on the non-clinical pharmacology and toxicology data spans all the documentation regarding the pharmacology and preclinical toxicity testing and, in the specific case of a PET imaging agent, pharmacokinetics and dosimetry estimates. Based on this documentation and data available on human experience, an overall risk and benefit assessment is provided to support the intended clinical trial.

**Figure 1:**
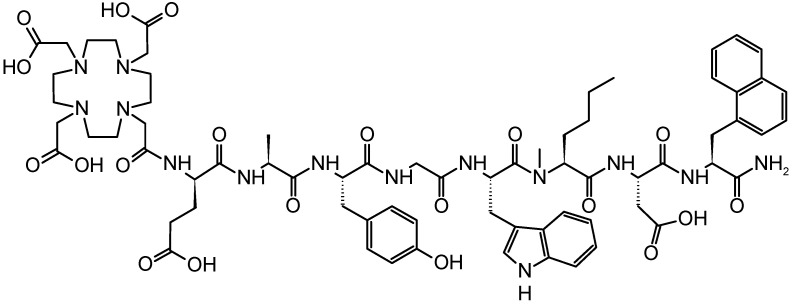
Chemical structure of DOTA-MGS5.

We have recently reported on a novel minigastrin (MG) analog targeting the cholecystokinin-2 receptor (CCK2R), expressed at high incidence in medullary thyroid carcinomas (MTC, 92%), small cell lung cancers (SCLC, 57%), astrocytomas (65%), stromal ovarian cancers (100%), as well as gastroenteropancreatic neuroendocrine tumors (NET, 22%) and other tumors [10,11,12,13]. The new MG analog was derived from DOTA-DGlu-Ala-Tyr-Gly-Trp-Met-Asp-Phe-NH_2_ (DOTA-MG11) by applying site-specific modifications in the C-terminal receptor binding sequence. These include replacement of the oxidation-sensitive methionine by N-methylated norleucine ((N-Me)Nle) and of the C-terminal phenylalanine by 1-naphthylalanine (1-Nal). The chemical structure of DOTA-DGlu-Ala-Tyr-Gly-Trp-(N-Me)Nle-Asp-1-Nal-NH_2_ (DOTA-MGS5) is presented in Figure 1. DOTA-MGS5 radiolabeled with different radiometals was efficiently stabilized against enzymatic degradation in vivo and showed a highly increased tumor uptake (>20% IA/g) combined with improved tumor-to-kidney ratio (4-6) in nude BALB/c mice xenografted with A431 cells transfected with human CCK2R (A431-CCK2R) [14].

The tumor targeting properties of DOTA-MGS5 are clearly superior to currently available MG analogs studied in the same mouse tumor model [14,15,16,17,18,19]. Two of these MG analogs, DOTA-(DGlu)_6_-Ala-Tyr-Gly-Trp-Met-Asp-Phe-NH_2_ radiolabeled with indium-111 (^111^In-CP04, formerly ^111^In-PP-F11; ClinicalTrials.gov Identifier: NCT03246659) and DOTA-(DGlu)_6_-Ala-Tyr-Gly-Trp-Nle-Asp-Phe-NH_2_ radiolabeled with lutetium-177 (^177^Lu-PP-F11N; ClinicalTrials.gov Identifier: NCT02088645) are currently being evaluated in clinical studies [20,21,22]. Preliminary dosimetry studies have demonstrated the feasibility of CCK2R targeting and identified the stomach as a possible dose limiting organ for therapeutic applications. In the vision of introducing the evaluation of the CCK2R status by high sensitivity PET imaging, we have focused our efforts on the clinical translation of ^68^Ga-labeled DOTA-MGS5.

In this work, the preparation of the new radiopharmaceutical [^68^Ga]Ga-DOTA-MGS5 (named ^68^Ga-DOTA-MGS5) using a cassette-based automated synthesis unit, as well as the preclinical testing in support of a phase I/IIa clinical trial application, is presented. The in vitro studies included the evaluation of receptor affinity and cell uptake in CCK2R expressing cell lines, biodistribution studies in mice for human dose extrapolations, and an extended single-dose toxicity study in rats.

## 2. Results and Discussion 

### 2.1. Radiolabeling and Quality Control

Experimental radiolabeling of DOTA-MGS5 with gallium-68 was carried out at molar activity of 10–30 GBq/µmole and resulted in a radiochemical yield of at least 95%. The radiolabeled peptide was used without further purification for in vitro studies. For animal experiments, the radiolabeled peptide was purified on a Sep-Pak^®^ Light tC18 cartridge (Waters, Milford, MA, USA) pretreated with 5 mL ethanol and 5 mL physiological saline. This procedure removed free and colloidal gallium-68 and ensured a high radiochemical purity of the radiolabeled peptide in the biodistribution and dosimetry studies. ^68^Ga-DOTA-MGS5 was eluted from the cartridge with 0.5 mL ethanol and diluted with phosphate buffered saline (PBS) to a final ethanol concentration of <2%. The solution was additionally supplemented with 10 µM bicarbonate to adjust the solution to a physiological pH, as well as a 20-fold molar excess of DTPA over the total peptide content to complex any free gallium-68.

**Figure 2:**
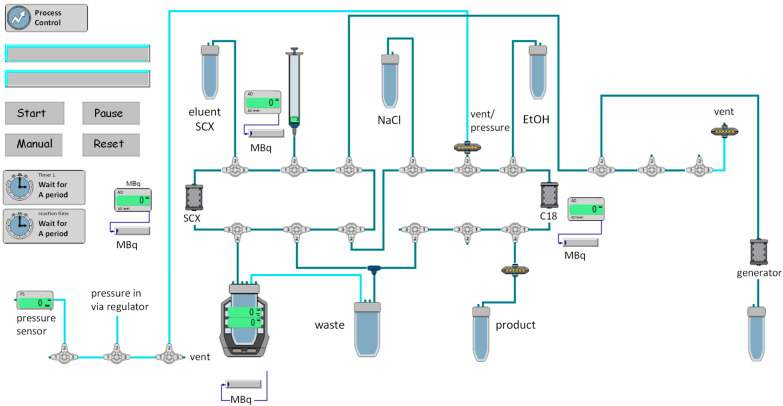
Scheme of the automated cassette-based production of ^68^Ga-DOTA-MGS5.

For the validation of the automated synthesis process, the standard radiolabeling process of other in-house prepared ^68^Ga-labeled radiopharmaceuticals was adapted for the labeling process of ^68^Ga-DOTA-MGS5. In Figure 2, the scheme of the process is shown. Four consecutive batches of ^68^Ga-DOTA-MGS5 were analyzed with a total radioactivity of 771–1231 MBq at the end of the production. The total synthesis time of ^68^Ga-DOTA-MGS5 from the start of the elution of the ^68^Ge/^68^Ga-generator to the radioactivity measurement of the end product was approximately 35 min. All four batches fulfilled the acceptance criteria for identification, radionuclide purity, radiochemical purity, peptide content, pH, ethanol content, sterility, and bacterial endotoxins (see Table 1). The product specifications were defined based on the current Ph. Eur. monographs “Chemical precursors for radiopharmaceutical preparations (2902)”, “Radiopharmaceutical Preparations (0125)”, “Gallium (^68^Ga) chloride solution for radiolabelling(2464)”, Gallium (^68^Ga) edotreotide injection (2482)”, Gallium (^68^Ga) PSMA-11 injection (3044)”, and “Fludeoxyglucose (^18^F) injection (1325)”. For the final product, a radioactivity concentration of 9–185 MBq/mL and a volume within the range of 7–12 mL was specified to cover different activities during the shelf life of the generator.

The identity of the final product was tested using high-performance liquid chromatography (HPLC) analysis and DOTA-MGS5 labeled with the stable isotope of gallium (Ga-DOTA-MGS5) as a reference standard. A relative retention time (RRT) of 0.9–1.1 was set for the determination of the proportion of the area of the principal peak due to ^68^Ga-DOTA-MGS5 relative to the total radioactivity of the chromatogram, and a limit of ≥0.91 was defined. Radiochemical impurities related to radiolytic side products were detectable at a retention time of ≥6 min. For the determination of these peptide-related radiochemical impurities arising from radiolytic processes during radiolabeling and purification, a RRT of 0.6–0.9 and 1.1–1.4 and a limit of ≤6% was set. In analogy to other ^68^Ga-labeled radiopharmaceuticals, the limit for the percentage of impurities determined by instant thin layer chromatography (iTLC) was set to ≤3%.

**Figure 3:**
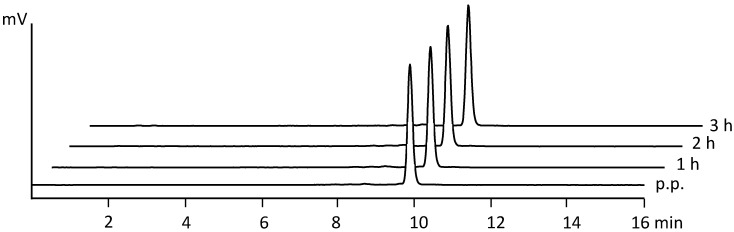
Radio-HPLC analysis of ^68^Ga-DOTA-MGS5 post preparation (p.p.) and at 1, 2, and 3 h EOS.

The radiochemical purity was calculated from the proportion of ^68^Ga-DOTA-MGS5 determined from the HPLC radiochromatogram (*T*) and the percentage of free or colloidal gallium-68 (*A*) determined by iTLC using the following formula: RCP = (100 – *A*) × *T*. The analysis of DOTA-MGS5, ^68^Ga-DOTA-MGS5, and other related substances in the UV trace was based on the sum of the areas of the peaks due to compounds with a RRT of not less than 0.8 and not more than 1.2 with reference to ^68^Ga-DOTA-MGS5. The total peak area was compared to the area of the principal peak of DOTA-MGS5 from calibration measurements, setting the limit to ≤50 µg/V. Post release tests included analysis of the ethanol (≤10%) and germanium-68 (≤0.001%) content, as well as bacterial endotoxin (<175 EU/V) and sterility testing. To evaluate the stability of ^68^Ga-DOTA-MGS5 during storage at room temperature, repetitive HPLC and iTLC analysis were performed to test the radiochemical purity over time. In Table 2, the tested acceptance criteria of the four consecutive batches are presented for the time points at 1, 2, and 3 h after preparation. The different radioactivity concentrations did not affect the overall radiochemical purity of the final product. A high radiochemical purity could be confirmed for ^68^Ga-DOTA-MGS5 formulated in physiological saline and <10% ethanol. After preparation, the percentage of free and colloidal gallium-68 did not exceed 1.4%, and the radiochemical purity was never below 92.2%. In Figure 3, the HPLC radiochromatograms for the different time points analyzed are shown for an exemplary batch. To guarantee a safe use of the product with radiochemical purity >91%, an expiry date of 2 h after end of synthesis (EOS) was set for the final product.

### 2.2. Non-Clinical Pharmacology and Toxicological Data

#### 2.2.1. Receptor Affinity Assay

**Figure 4:**
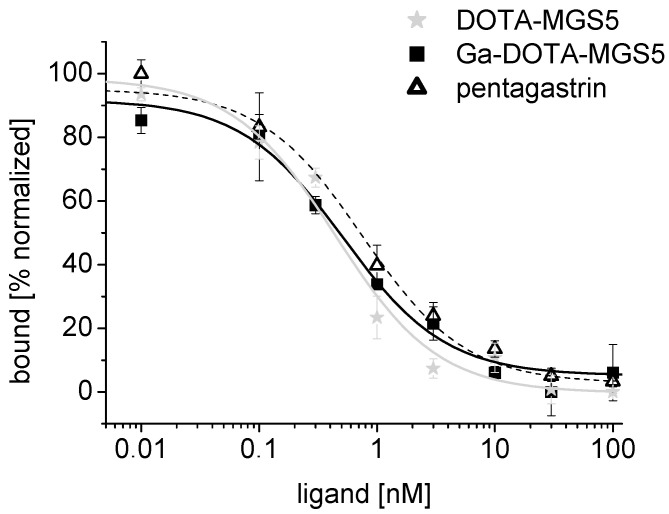
Exemplary binding curves for DOTA-MGS5, Ga-DOTA-MGS5, and pentagastrin.

In competition assays against [^125^I][3-iodo-Tyr^12^,Leu^15^]gastrin-I, a high binding affinity to CCK2R was confirmed on A431-CCK2R cells with values of 0.5 ± 0.1 nM (*n* = 3) for DOTA-MGS and 0.5 ± 0.1 nM for Ga-DOTA-MGS5 (*n* = 1). Pentagastrin used for comparison showed an IC_50_ value of 0.9 ± 0.1 nM (*n* = 3). In Figure 4, exemplary binding curves are presented for all compounds tested.

#### 2.2.2. Cell Uptake in CCK2R-Expressing Cell Lines

**Figure 5:**
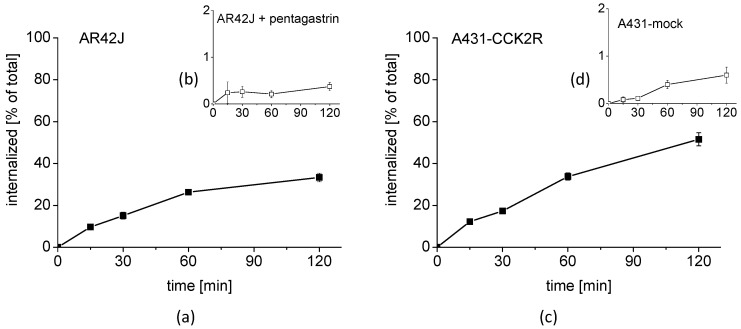
Cell uptake of ^68^Ga-DOTA-MGS5 in (**a**) AR42J cells and (**b**) AR42J cells co-incubated with pentagastrin, as well as (**c**) A431-CCK2R cells and (**d**) A431-mock cells for up to 2 h after incubation.

A high cell uptake of ^68^Ga-DOTA-MGS5 was found in two cell lines with CCK2R expression. In AR42J cells with physiological expression of rat CCK2R, a cell uptake of 33.3 ± 2.1% after 2 h incubation was observed, which was effectively blocked by co-incubation with 1 µM pentagastrin (Figure 5a,b). In internalization assays with A431-CCK2R, a somewhat higher uptake value of 51.5 ± 3.1% was observed for the same time point (Figure 5c). Receptor specificity was confirmed by parallel cell uptake studies in A431-mock cells without CCK2R expression showing a very low unspecific uptake of 0.6 ± 0.2 (Figure 5d).

The cell uptake compares well with previous studies performed with DOTA-MGS5, in which a very similar cell uptake of 50–60% was observed in A431-CCK2R cells for DOTA-MGS5 radiolabeled with indium-111, gallium-68, and lutetium-177 [14].

The cell uptake of ^68^Ga-DOTA-MGS5 was superior to other CCK2R-specific radioligands currently investigated in clinical studies. For the time point of 1 h incubation, a cell internalization of ~20% was reported for ^177^Lu-DOTA-PP-F11N in A431-CCK2R cells [23]. For ^111^In-CP04, a cell uptake of 14.4 ± 0.8 % was reached in AR42J cells for the time point of 4 h after incubation [24].

#### 2.2.3. Interaction with Other Receptors

**Figure 6:**
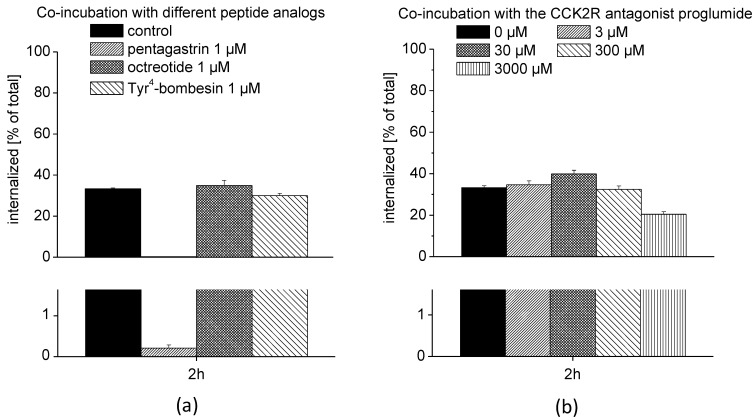
Cell uptake studies with ^68^Ga-DOTA-MGS5 on AR42J cells: (**a**) Co-incubation with different peptide analogs specific for CCK2R, SSTR, and GRPR; (**b**) co-incubation with increasing concentrations of the CCK2R antagonist proglumide.

AR42J cells display neuroendocrine properties and express somatostatin receptors (SSTR) and gastrin releasing peptide receptors (GRPR) in addition to CCK2R [25,26,27]. To test for a possible interaction with other G-protein coupled receptors, AR42J cells were co-incubated with ^68^Ga-DOTA-MGS5 and different peptide analogs specific for CCK2R, SSTR, and GRPR. As shown in Figure 6a, the cell uptake of ^68^Ga-DOTA-MGS5 could be efficiently blocked by co-incubation with pentagastrin, reducing the cell uptake to values of 0.21 ± 0.08% when compared with the control uptake without pentagastrin (33.2 ± 2.1%). No blockage of the cell internalization was obtained by co-incubation with octreotide (34.9 ± 2.5%) and Tyr^4^-bombesin (29.9 ± 1.1%), confirming specific interaction with CCK2R. In addition to blocking of the CCK2R interaction using the agonist pentagastrin, the effect of increasing concentrations of the CCK2R antagonist proglumide on the receptor-specific cell internalization was also investigated (see Figure 6b). Only at a very high concentration of 3000 µM proglumide was a considerable reduction of the cell uptake achieved (20.4 ± 1.3%). Partial displacement of ^68^Ga-DOTA-MGS5 from the receptor when co-incubated with elevated concentrations of the antagonist proglumide suggests a possible interaction with different conformational states of the receptor or a larger number of binding sites.

#### 2.2.4. Biodistribution and Dosimetry Studies in BALB/c Mice

**Figure 7:**
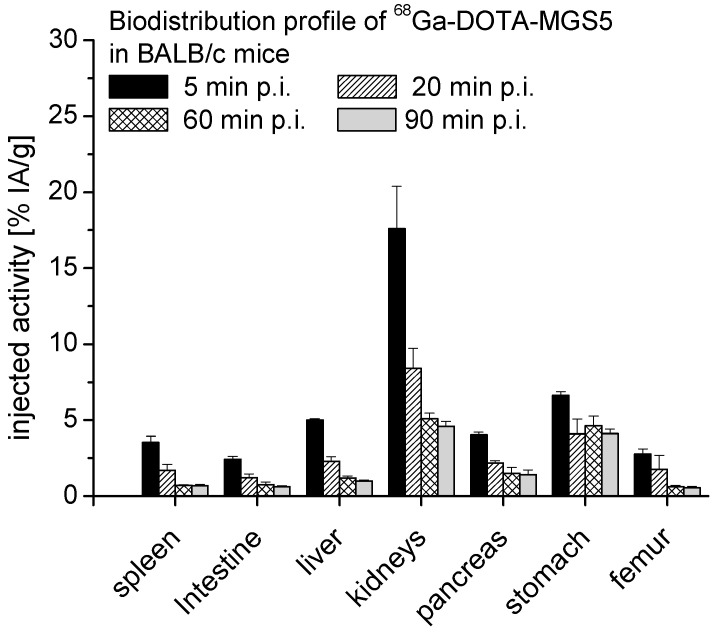
Biodistribution of ^68^Ga-DOTA-MGS5 in BALB/c mice at 5, 20, 60, and 90 min p.i. (*n* = 12).

The biodistribution profile of ^68^Ga-DOTA-MGS5 in female BALB/c mice was evaluated for the time points of 5, 20, 60, and 90 min post injection (p.i.). A low unspecific uptake was observed in most tissues, together with fast blood clearance and low kidney retention. The whole body activity determined for the animals was 81.8 ± 2.5% IA at 5 min p.i. and declined to 11.4 ± 1.7% IA at 90 min after injection. Uptake levels of 17.6 ± 3.0% IA/g for blood and 17.6 ± 2.8% IA/g for kidneys were observed at 5 min p.i. and rapidly cleared to values of 0.9 ± 0.1% IA/g in blood and 4.6 ± 0.3% IA/g in kidneys at 90 min after injection. In CCK2R expressing organs, a somewhat higher uptake of 4.1–6.6% IA/g was observed in the stomach, whereas a lower activity was found in the pancreas with values of 1.4–4.0% IA/g. The uptake values in the liver were in the range of 1.0–5.0% IA/g, and for the intestine, uptake levels of 0.6–2.4 % IA/g were found. A bone uptake of 0.6–2.8% IA/g was determined for the excised part of the femur. In Figure 7, the uptake values for these main target organs are summarized, whereas in Table 3, the uptake values in all examined tissues are given.

Dosimetry calculations were based on the uptake values found for the lung, heart, muscle, spleen, liver, kidneys, pancreas, and stomach. For extrapolation of the biodistribution data from mice to humans, the studied time points were multiplied with a factor of 10 to account for the faster kinetics in mice versus humans. A similar uptake per organ was assumed for mice with a mean body weight of 17.5 g, which was derived from the group of animals studied at 5 min p.i., and for humans with a standard body weight of 73.7 kg, which is used in the Medical Internal Radiation Dosimetry (MIRD) system of dose calculation [28].

When extrapolating the biodistribution data from mice to humans, a whole-body effective dose of 0.0102 mSv/MBq for men and of 0.0131 mSv/MBq for women was calculated. In Table 4, a summary of the calculated organ absorbed doses is given. At a maximum injected activity of 185 MBq, a whole-body radiation dose of 1.9–2.4 mSv can be anticipated for a PET examination with ^68^Ga-DOTA-MGS5. The radiation exposure from the radioactive substance is in a similar range of a low-dose CT scan of 3–6 mSv [29]. Due to the potential hazards of ionizing radiation, the dose of the PET/CT scan should be kept as low as possible to reduce the radiation exposure to the patient but still ensure good image quality [30]. An injected activity of 185 MBq is in line with other state of art PET examinations with ^68^Ga-labeled ligands [31]. An effective radiation dose of 2.4 mSv was reported for an injected dose of 150 MBq for ^68^Ga-PSMA-11 and of 2.1 mSv for an injected dose of 100 MBq for ^68^Ga-DOTA-TOC [32,33].

Based on the uptake values observed in the animal biodistribution study, the kidney and stomach are expected to receive the highest organ radiation doses. This has no implication for the planned diagnostic application but needs to be considered for a potential therapeutic use. During peptide receptor radionuclide therapy (PRRT) with radiolabeled peptides, fractionation of therapy is used to reduce the radiation induced toxicity to dose limiting organs. A cumulative absorbed dose of 27 Gy is generally accepted for the kidneys, whereas for the stomach, a maximum dose of 50 Gy is generally accepted in radiation therapy [34,35].

#### 2.2.5. Extended Single-Dose Toxicity Study in Rats

In the preclinical evaluation of the safety profile of DOTA-MGS5, a targeted approach was followed for the 14-day extended single-dose toxicity study conducted in male Wistar rats. Instead of performing the study in animals of both sexes at a single dose level, the non-clinical evaluation was confined to male animals, including, however, three different dose levels based on mg/kg scaling and body surface scaling (applying a factor of 6.2). Considering a maximum dose of 50 µg administered to patients and an exposure margin of 100-fold the clinical dose, a dose level of 0.5 mg/kg body weight was calculated for the study and used as medium dose. Two additional dose levels of 0.1 mg/kg body weight and 2.5 mg/kg body weight were selected to allow a more accurate evaluation of the safety profile. Based on the preclinical biodistribution studies, the stomach, pancreas, and kidneys were identified as the main target organs. In addition to these organs, the liver and colon as potential additional routes of excretion, as well as spleen and sternum (including bone marrow), were also included in the histopathological evaluation.

In the performed study, no treatment-related mortality or clinical symptoms occurred after intravenous administration of DOTA-MGS5 at the three different dose levels of 0.1, 0.5, and 2.5 mg/kg BW until the end of the observation periods of the study. Furthermore, no DOTA-MGS5 related effects on body weight development, food consumption, urine, or coagulation could be determined. No treatment-related necropsy findings or abnormalities concerning organ weights were observed in any of the dose groups at the end of the observation periods. Only minor effects on hematologic parameters and clinical biochemistry were observed, which were not considered as toxicologically relevant. All animals showed no signs of toxicity, and no findings were reported in the histopathological evaluation. A NOAEL (no observed adverse effect level) of 2.5 mg/kg body weight was established from the study for the single administration of DOTA-MGS5. When considering body surface scaling from rats to humans, the NOAEL of 2.5 mg/kg body weight translates to a HED (human equivalent dose) of 0.4 mg/kg body weight. By applying a safety factor of 100, a maximum dose of 4 µg/kg body weight was established for human subjects receiving the initial clinical dose. For the first PET/CT examinations with ^68^Ga-DOTA-MGS5, a maximum dose of 50 µg could be established as clinical dose, which remains well below the maximum dose of 4 µg/kg body weight.

## 3. Materials and Methods

### 3.1. Radiolabeling Procedure and Quality Control 

#### 3.1.1. Reagents for Radiolabeling

For the in-house preparation of the solutions used in the radiolabeling process, either reagents of Ph. Eur. grade, such as sodium acetate trihydrate (1.06267, Merck, Darmstadt, Germany) and sodium chloride (1.06406, Merck, Darmstadt, Germany), or reagents with high purity grade for trace analysis, including glacial acetic acid (83876.270, VWR, Leuven, Belgium), 30% hydrochloric acid (1.01514, Merck, Darmstadt, Germany), and water (14211, Sigma-Aldrich, Steinheim, Germany), were used. All other solutions used in the formulation process, including 95% ethanol, water for injection, 50% ethanol, and physiological saline, were from sterile pharmaceutical formulations for parenteral application.

The precursor, DOTA-MGS5, was obtained in GMP quality from piCHEM (Raaba-Grambach, Austria) in lyophilized aliquots of 250 μg in 2 mL ISO clear type I tubular glass vials. The aliquots were dissolved in 50 µL 95% ethanol and 200 µl water for injection at a concentration of 1 mg/mL. Using this solution, a calibration curve was generated for the quantification of the peptide content based on the integration of the UV signal at 280 nm of the HPLC chromatogram.

[^68^Ga]GaCl_3_ used in the validation process was obtained from a chemical grade ^68^Ge/^68^Ga generator (IGG100 generator Eckert & Ziegler Radiopharma GmbH, Berlin, Germany) eluted with 0.1 N HCl solution (Rotem Industries Ltd., Beer-Sheva, Israel) or a pharmaceutical grade ^68^Ge/^68^Ga generator (GalliaPharm^®^, Eckert & Ziegler Radiopharma GmbH, Berlin, Germany) eluted with sterile ultrapure 0.1 N HCl solution (Eckert & Ziegler Radiopharma GmbH, Berlin, Germany), both with maximal theoretical activity of 1850 MBq.

#### 3.1.2. Labeling of DOTA-MGS5 with Gallium-68

For preclinical testing, radiolabeling with gallium-68 was performed using 10–20 µL DOTA-MGS5 solution (corresponding to 10–20 µg) in a low protein binding Eppendorf tube^®^ (Eppendorf AG, Hamburg, Germany) together with 400–500 µL [^68^Ga]GaCl_3_ eluate (70–260 MBq in 0.1 M HCl). The solution was adjusted to pH 3.8–4.0 with 40–50 µL ~1.14 M sodium acetate solution and incubated at 95 °C for 10 min. For animal studies, the radiolabeling solution was purified by solid-phase extraction.

For clinical use, radiolabeling of the precursor DOTA-MGS5 with gallium-68 was performed in a clean-room class C environment using an automated synthesis module (Modular-Lab PharmTracer^®^, Eckert & Ziegler Eurotrope GmbH, Berlin, Germany), a sterile disposable cassette (C4-GA-PEP, Eckert & Ziegler Eurotrope GmbH, Berlin, Germany), and a set of commercially available accessories or in-house prepared reagents. Before starting the automated synthesis, the reaction vial of the cassette was pre-loaded with 50 µL DOTA-MGS5 (1 mg/mL, 19% ethanol) and 1.5 mL of a 1.45 M sodium acetate buffer pH 3.8–4.1 containing 200 µL 95% EtOH. Three milliliters of 4.9 M NaCl/0.14 M HCl solution was dispensed in the eluent vial. A 20 mL vial with 50% ethanol and a 50 mL vial with physiological saline were mounted and the product vial connected via a Millex-GV 0.22 µm low protein binding sterile filter (SLGV033RS, Merck Millipore, Carrigtwohill, Ireland) and a cannula to the product vial, as well as the generator line and the waste line connected (see Figure 1). All liquid transfers were performed via the dispenser syringe of the cassette and the integrated syringe module of the synthesis unit. For the validation process, gallium-68 was obtained from a ^68^Ge/^68^Ga-generator of pharmaceutical or chemical grade. [^68^Ga]GaCl_3_ was eluted from the generator with up to 9 mL 0.1 M hydrochloric acid and passed through the SCX cartridge, followed by elution of gallium-68 from the cartridge with approximately 0.8 mL eluent solution and transfer of the solution to the reaction vial. Radiolabeling was performed at 95 °C for 7 min. For purification, the reaction mixture was passed through the Sep-Pak^®^ Light C18 cartridge, preconditioned with 50% ethanol and physiological saline during the radiolabeling step. ^68^Ga-DOTA-MGS5 was eluted from the cartridge using 1 mL 50% ethanol, transferred to the product vial through the sterile filter and diluted with 7.5 mL physiological saline, resulting in the final injection solution.

#### 3.1.3. Quality Control of ^68^Ga-DOTA-MGS5

The specifications and analytical procedures for the quality control of ^68^Ga-DOTA-MGS5 were derived from the Ph. Eur. monographs.

Gallium-68. Radioactivity measurements were performed using a VDC-405 dose calibrator (Veenstra Instruments, Joure, Netherlands). Identification of the radionuclide prior to release was carried out by gamma ray spectrometry (GabiStar, Raytest, Straubenhardt, Germany) and half-life calculation. Radionuclide purity with regard to germanium-68 was quantitatively determined after release following a minimum decay time of at least 48 h.

DOTA-MGS5, ^68^Ga-DOTA-MGS5, and other related substances. Radiochemical purity of ^68^Ga-DOTA-MGS5 was analyzed using HPLC and iTLC. For HPLC analysis, an UltiMate 3000 chromatography systems (Dionex, Germering, Germany), consisting of a HPLC pump, an autosampler, a variable UV-detector (UV-VIS at λ = 280 nm), and a radiodetector (GabiStar, Raytest, Straubenhardt, Germany), was used together with different reversed-phase analytical HPLC columns and water/0.1% TFA (solvent A) and ACN/0.1%TFA (solvent B) as mobile phases. For experimental labeling, a Phenomenex Jupiter 4 µm Proteo 90 Å C12 column, 250 × 4.6 mm (00G-4396-E0, Phenomenex Ltd., Aschaffenburg, Germany) with flow rate of 1 mL/min was used together with the following gradient: 0–3 min 10% B, 3–18 min 10–55% B, 18–20 min 55–80% B, 20–21 min 80–10% B, 21–25 min 10% B. For the validation process of the automated synthesis, the HPLC gradient was adapted using an ACE 3 μm C18 column, 150 × 3 mm (ACE-111-1503, Advanced Chromatography Technologies Ltd., Aberdeen, Scotland) with flow rate 0.6 mL/min together with the following gradient: 0–2 min 28% B, 2–12 min 28–55% B, 12–12.1 min 55–28% B, 12.1–16 min 28% B. Chromeleon Dionex Software (Version 7.2.9) was used as analyzing software. DOTA-MGS5 complexed with the stable isotope of gallium was used as a reference standard to test the identity of ^68^Ga-DOTA-MGS5. From the radiochromatogram, the proportion of the peak corresponding to ^68^Ga-DOTA-MGS5 in relation to the total area of the radioactivity peaks was calculated. For this purpose, the whole radiochromatogram was integrated from the center of the baseline noise. The peptide content was calculated by baseline-to-baseline integration of the peaks related to the chemical precursor (peak areas below the limit of detection were disregarded) in the UV chromatogram. A limit test for the peptide content was performed considering the sum of the peak areas corresponding to DOTA-MGS5 and metal complexes thereof observed in the UV trace. The peptide content was calculated based on a calibration curve. iTLC using Agilent iTLC-SG, 9×1 cm chromatography paper, and a 1:1 mixture of 1 M ammonium acetate and methanol as mobile phase was used for the evaluation of the radionuclide incorporation. A Scan-RAM radio-TLC scanner with a PS Plastic/PMT detector (LabLogic Systems, Sheffield, UK) was used to determine the percentage of impurities migrating with a retardation factor lower than 0.2.

pH. The pH of the final injectable solution was determined by use of pH indicator strips (1.09526.0003, Universal indicator, Merck).

Ethanol. The analysis of the ethanol content was performed on a GC-2010 Plus gas chromatograph with AOC-20i autosampler (Shimadzu, 2100 Korneuburg, Austria), equipped with a Phenomenex Zebron ZB-624 column (7KM-G005-31), using a flow rate of 3 mL/min and a FID temperature of 260 °C.

Sterility. At the end of the synthesis process, a filter integrity test was performed for the sterile filter using a specific program of the automated synthesis module prior to release. In addition, sterility testing according to Ph. Eur. was performed post release.

Bacterial endotoxins. Bacterial endotoxin testing was performed prior to release using an Endosafe PTS Reader and PTS20F Limulus Amebocyte Lysate (LAL) test cartridges (Charles River Labortatories, Charleston, SC, USA).

Stability. To evaluate the shelf-life of the final formulation, the radiochemical purity was assessed by radio-HPLC and iTLC analysis for up to 3 h post preparation (EOS). In addition, the peptide content was calculated from the UV-trace.

### 3.2. Non-Clinical Pharmacology and Toxicology Data

#### 3.2.1. Cell Lines with CCK2R Expression

The receptor interaction of ^68^Ga-DOTA-MGS5 was tested in two different cell lines. The A431 human epidermoid carcinoma cell line stably transfected with the plasmid pCR3.1 containing the full coding sequence for the human CCK2R, as well as the same cell line transfected with the empty vector alone, was kindly provided by Dr. Luigi Aloj [36]. AR42J rat pancreatic tumor cells expressing rat CCK2R were obtained from European Collection of Authenticated Cell Cultures (ECACC, Salisbury, UK). A431-CCK2R and A431-mock cells were cultured in Dulbecco’s Modified Eagle Medium (DMEM) and AR42J cells were cultured in RPMI-1640 medium, both supplemented with 10% (*v*/*v*) fetal bovine serum and 5 mL of a 100x penicillin–streptomycin–glutamine mixture at 37 °C in a humidified 95% air/5% CO_2_ atmosphere. Media and supplements were purchased from Invitrogen Corporation (Lofer, Austria).

#### 3.2.2. Receptor Affinity Assay

The CCK2R affinity of DOTA-MGS5 and Ga-DOTA-MGS5 was tested in competition assays against [^125^I][3-iodo-Tyr^12^,Leu^15^]gastrin-I on A431-CCK2R cells. Pentagastrin was used for comparison. Binding assays on A431-CCK2R cells were carried out as previously described [37]. The cells were incubated with increasing concentrations of the peptide conjugates and [^125^I][3-iodo-Tyr^12^,Leu^15^]gastrin-I (~20,000 cpm) for 1 h at RT. Half-maximum inhibitory concentration (IC_50_) values were calculated after nonlinear regression using Origin software (Microcal Origin 6.1, Northampton, MA, USA). For the graphical presentation of exemplary binding curves, data were normalized from 0 to 100.

#### 3.2.3. Receptor-Specific Cell Uptake

The cells were seeded at a density of 1.0 × 10^6^ for A431-CCK2 and 1.5 × 10^6^ for AR42J per well in 6-well plates (Greiner Labortechnik, Kremsmuenster, Austria) and grown for 48 h until reaching almost confluence. On the day of the experiment, the medium was replaced by 1.2 mL of fresh medium supplied with 1% (*v*/*v*) fetal bovine serum and ~50,000 cpm of ^68^Ga-DOTA-MGS5 in 300 µL PBS/0.5% BSA, reaching a total volume of 1.5 mL, corresponding to a final concentration of 0.4 nM of total peptide. After 15, 30, 60, and 120 min of incubation, the cell uptake was interrupted by removal of the medium and rinsing twice with 1 mL PBS/0.5% BSA. Thereafter, the cells were incubated twice at ambient temperature with acid wash buffer (50 mM glycine buffer, pH 2.8, 0.1 M NaCl) for 5 min to remove the membrane-bound radioligand. Finally, the cells were lysed by treatment in 1 N NaOH and collected (internalized radioligand fraction). All fractions were counted in the gamma-counter (2480 Wizard^2^ 3”, PerkinElmer Life Sciences and Analytical Sciences, formerly Wallac Oy, Turku, Finland), and mean values were calculated. The cell uptake was expressed in relation to the total radioactivity added to the cells (% of total). Non-specific binding was evaluated by either blocking with 1 µM of pentagastrin or using A431-mock cells.

#### 3.2.4. Interaction with Other Receptors

The interaction with other receptors was evaluated in AR42J cells. In addition to expression of CCK2R, AR42J cells also express SSTR and GRPR. The cells were seeded at a density of 1.5 × 10^6^ in 6-well plates and grown for 48 h. On the day of the experiment, the cells were incubated with ^68^Ga-DOTA-MGS5 (0.4 nM) for 2 h in the absence or presence of blocking concentrations of different peptide derivatives. To block the interaction with CCK2R the agonist pentagastrin (B1636-25MG, Sigma-Aldrich, Darmstadt, Germany) (1 µM) was used. The interaction with SSTR was blocked using octreotide (Sandostatin^®^ Novartis Pharma GmbH, Wien, Austia) (1 µM) and the interaction with GRPR using Tyr^4^-bombesin (B5397-1MG, Sigma-Aldrich, Darmstadt, Germany) (1 µM). Blockage of the CCK2R interaction was additionally confirmed by co-incubation with increasing concentrations of the antagonist proglumide (095m4609v, Sigma-Aldrich, Darmstadt, Germany) (3–3000 µM). The cells were treated as described above and all fractions counted in the gamma-counter to determine the internalized radioligand fraction.

#### 3.2.5. Pharmacokinetics and Dosimetry in Animals

Biodistribution studies evaluating the pharmacokinetics and tissue distribution of ^68^Ga-DOTA-MGS5 were performed in 6-week-old female BALB/c mice (*n* = 12). Groups of three mice for each time point were injected intravenously via a lateral tail vein with ~1 MBq of ^68^Ga-DOTA-MGS5 corresponding to 20 pmol peptide. The mice were euthanized by cervical dislocation 5, 20, 60, and 90 min after injection and different tissues (blood, lung, heart, muscle, spleen, intestine, liver, kidneys, pancreas, stomach, and femur) were removed, weighed, and their radioactivity measured in a gamma-counter together with a standard and the residual body. Results were expressed as percentage of injected activity per organ (% IA) and per gram tissue (% IA/g).

For dosimetry estimations, based on the data obtained from the biodistribution study, the injected dose per organ found for mice was extrapolated to humans using the following equation: % IA per organ in humans = [% IA/g in mice × mass of the mice (kg)] × mass of a human organ (g) divided by the total body mass (kg). The activity delivered to the red bone marrow was derived from the activity found in the measured bone sample [38]. The bladder activity was calculated by subtraction of the total activity found in the animals for each time point from the total activity administered. Blood and intestine were included in the activity of the remainder body. Based on these data, the expected absorbed dose per injected activity in humans was calculated using Olinda-EXM (Version 1.1, Vanderbilt University).

#### 3.2.6. Extended Single-Dose Toxicity Study in Rats

The safety of the intravenous injection was tested for unlabeled DOTA-MGS5 in a GLP-compliant 14-day extended single-dose intravenous toxicity study in rats using the intravenous route of administration. The study was designed following a targeted microdose approach based on the ICH guideline M3 (R2) on non-clinical safety studies for the conduct of human clinical trials and marketing authorization for pharmaceuticals (EMA/CPMP/ICH/286/1995, December 2009), as well as the draft guideline on the non-clinical requirements for radiopharmaceuticals (EMA/CHMP/SWP/686140/2018, 15 November 2018). The study was conducted in a GLP-compliant laboratory (BSL BIOSERVICE Scientific Laboratories Munich GmbH, Planegg/Munich, Germany) using 60 6–7-week-old male healthy Wistar rats (Crl: WI(Han), Full Barrier, Charles River, Sulzfeld, Germany). The animals were divided in four groups of 15, including three dose groups and one control group observed over a period of 2 and 14 days after single intravenous administration. Phosphate buffered saline (SC-01-PbS, ABX advanced biochemical compounds, Radeberg, Germany) was used as a vehicle to dissolve and administer DOTA-MGS5. Control animals were handled identically but received only PBS by single intravenous injection. During and after administration, the animals were observed precisely each day for signs of toxicity. Body weight and food consumption was determined on a weekly basis. Ten animals of each group were euthanized one day after administration and then examined macroscopically and histopathologically. Hematologic parameters and blood biochemistry was investigated on day 2 and day 14. In order to allow a detection of possible delayed occurrence, five animals per group were observed for a period of 14 days following administration and examined macroscopically thereafter. Before or during necropsy, urine was collected for further analyses, and a defined set of organs was weighed and preserved. The histopathological evaluation confined to selected tissues was performed for five animals per group for each observation period.

## 4. Conclusions

^68^Ga-DOTA-MGS5 is a promising new radiopharmaceutical for the evaluation of the CCK2R status in patients with CCK2R-related malignancies. The standardized production using a cassette-based automated synthesis module and the performed preclinical testing support a first pilot study evaluating the safety of administration and biodistribution of ^68^Ga-DOTA-MGS5 in patients with advanced NET. Furthermore, the visualization of tumor lesions will be investigated, and preliminary dosimetry will be performed. Patients with advanced MTC and patients with gastroenteropancreatic and bronchopulmonary NET will be included in this first exploratory clinical study.

Most of the previous clinical studies with radiolabeled MG analogs were performed using conventional scintigraphic imaging techniques based on radioligands labeled with indium-111 and technetium-99m. So far, only in two patients has PET imaging with a ^68^Ga-labeled CCK2R targeting peptide analog been reported [39,40]. The availability of high sensitivity PET/CT using ^68^Ga-DOTA-MGS5 for tumor detection will have a significant clinical impact. In addition, DOTA-MGS5 also has great potential for therapeutic application when radiolabeled with beta emitting radioisotopes, such as yttrium-90 and lutetium-177.

## Figures and Tables

**Table 1:** Batch analysis table of four consecutive batches of ^68^Ga-DOTA-MGS5.

Parameter	Method	Acceptance Criteria	Results (*n* = 4)
Appearance	Visual inspection	Clear, colorless solution, free of visible particles	conforms
pH	Indicator strip	4.0–8.0	6.0
Volume	Graduated vial	8.5 (range 7–12 mL)	8.5 mL
Radioactivity concentration	MBq/mL	9–185 MBq/mL	112 ± 20 MBq/mL
Radionuclide identity	Gamma-ray spectrometry	511 keV; 1022 keV	conforms
Radionuclide identity	Half-life	62–74 min	68.3 ± 0.3 min
Identity of ^68^Ga-DOTA-MGS5 (comparison with reference)	HPLC	RRT 0.9–1.1	conforms
Proportion of ^68^Ga-DOTA-MGS5	HPLC (*T*)	not specified	0.949 ± 0.012
Radiochemical impurities with RRT 0.6–0.9 and 1.1–1.4	HPLC	≤6%	4.3 ± 0.8%
Free or colloidal gallium-68 (retardation factor <0.2)	iTLC (*A*)	≤3%	0.9 ± 0.5%
Radiochemical purity	RCP = (100 – *A*) × *T*	≥91%	94.1 ± 1.3%
DOTA-MGS5, ^68^Ga-DOTA-MGS5 and related substances (RRT 0.8–1.2)	HPLC	≤50 µg/V	<40 µg
Ethanol content	Gas chromatography	≤10 % (*v*/*v*)	6.2 ± 0.5%
Radionuclide purity	Gamma-ray spectrometry	Ge-68: ≤0.001% (after ≥48 h)	conforms
Bacterial Endotoxins	LAL test	≤175 IU/V	<3 IU/V
Sterility	Ph. Eur.	sterile	conforms

**Table 2:** Stability testing of ^68^Ga-DOTA-MGS5 up to 3 h after preparation.

Stability Testing	Acceptance Criteria	1 h	2 h	3 h
Free or colloidal gallium-68 (retardation factor <0.2)	≤3%	0.5 ± 0.3	0.5 ± 0.4	0.6 ± 0.2
Radiochemical purity	≥91%	94.3 ± 1.2	92.9 ± 1.0	92.9 ± 1.5
DOTA-MGS5, ^68^Ga-DOTA-MGS5 and related substances (RRT 0.8–1.2)	≤50 µg/V	<40	<40	<40

**Table 3:** Uptake values obtained from biodistribution studies with ^68^Ga-DOTA-MGS5 (0.2–0.5 MBq, 20 pmol peptide) at 5, 20, 60, and 90 min p.i. in female BALB/c mice; values expressed as % IA/g (mean ± SD, *n* = 3).

	^68^Ga-DOTA-MGS5 % IA/g 5 min p.i.	^68^Ga-DOTA-MGS5 % IA/g 20 min p.i.	^68^Ga-DOTA-MGS5 % IA/g 60 min p.i.	^68^Ga-DOTA-MGS5 % IA/g 90 min p.i.
organ	mean ± sd	mean ± sd	mean ± sd	mean ± sd
blood	17.58 ± 3.01	6.76 ± 1.17	1.99 ± 0.22	0.94 ± 0.08
lung	8.51 ± 2.33	3.78 ± 0.53	1.10 ± 0.11	0.59 ± 0.10
heart	5.11 ± 0.07	2.24 ± 0.57	0.70 ± 0.16	0.35 ± 0.02
muscle	2.89 ± 0.18	1.03 ± 0.21	0.49 ± 0.19	0.18 ± 0.04
spleen	3.53 ± 0.43	1.70 ± 0.38	0.71 ± 0.05	0.69 ± 0.09
intestine	2.42 ± 0.21	1.22 ± 0.25	0.75 ± 0.17	0.62 ± 0.08
liver	5.02 ± 0.06	2.28 ± 0.31	1.21 ± 0.12	1.00 ± 0.06
kidneys	17.60 ± 2.79	8.41 ± 1.33	5.11 ± 0.33	4.60 ± 0.32
pancreas	4.03 ± 0.19	2.17 ± 0.16	1.50 ± 0.38	1.42 ± 0.30
stomach	6.63 ± 0.23	4.09 ± 0.98	4.63 ± 0.65	4.12 ± 0.30
femur	2.77 ± 0.34	1.75 ± 0.93	0.62 ± 0.09	0.55 ± 0.09

**Table 4:** Expected organ absorbed doses of ^68^Ga-DOTA-MGS5 in humans based on extrapolation of the biodistribution data from mice to humans.

Target Organ	Male mGy/MBq	Female mGy/MBq
adrenals	8.69 × 10^−3^	1.15 × 10^−2^
brain	7.67 × 10^−3^	9.93 × 10^−3^
breasts	7.15 × 10^−3^	9.40 × 10^−3^
gallbladder wall	8.93 × 10^−3^	1.11 × 10^−2^
LLI wall	8.72 × 10^−3^	1.14 × 10^−2^
small intestine	9.05 × 10^−3^	1.09 × 10^−2^
stomach wall	1.13 × 10^−2^	1.47 × 10^−2^
ULI wall	8.90 × 10^−3^	1.16 × 10^−2^
heart wall	1.12 × 10^−2^	1.43 × 10^−2^
kidneys	2.96 × 10^−2^	3.79 × 10^−2^
liver	1.08 × 10^−2^	1.37 × 10^−2^
lungs	1.57 × 10^−2^	1.99 × 10^−2^
muscle	7.00 × 10^−3^	8.86 × 10^−3^
ovaries	-	1.13 × 10^−2^
pancreas	9.86 × 10^−3^	1.24 × 10^−2^
red marrow	8.56 × 10^−3^	1.05 × 10^−2^
osteogenic cells	1.22 × 10^−2^	1.74 × 10^−2^
skin	6.67 × 10^−3^	8.76 × 10^−3^
spleen	8.29 × 10^−3^	1.05 × 10^−2^
testes	7.66 × 10^−3^	-
thymus	7.97 × 10^−3^	1.06 × 10^−2^
thyroid	7.88 × 10^−3^	9.91 × 10^−3^
urinary bladder wall	1.47 × 10^−2^	1.79 × 10^−2^
uterus	-	1.13 × 10^−2^
total body	9.78 × 10^−3^	1.25 × 10^−2^

LLI = lower large intestine, SI = small intestine, ULI = upper large intestine.

## Data Availability

Data are contained within the article.

## References

[1] Fallahi B., Manafi-Farid R., Eftekhari M., Fard-Esfahani A., Emami-Ardekani A., Geramifar P., Akhlaghi M., Hashemi Taheri A.P., Beiki D. (2019). Diagnostic efficiency of (68)Ga-DOTATATE PET/CT as compared to (99m)Tc-Octreotide SPECT/CT and conventional morphologic modalities in neuroendocrine tumors. Asia Ocean J. Nucl. Med. Biol..

[2] Gabriel M., Decristoforo C., Kendler D., Dobrozemsky G., Heute D., Uprimny C., Kovacs P., Von Guggenberg E., Bale R., Virgolini I.J. (2007). 68Ga-DOTA-Tyr3-octreotide PET in neuroendocrine tumors: Comparison with somatostatin receptor scintigraphy and CT. J. Nucl. Med. Off. Publ. Soc. Nucl. Med..

[3] Kwekkeboom D.J., Kam B.L., van Essen M., Teunissen J.J.M., van Eijck C.H.J., Valkema R., de Jong M., de Herder W.W., Krenning E.P. (2010). Somatostatin receptor-based imaging and therapy of gastroenteropancreatic neuroendocrine tumors. Endocr. Relat. Cancer.

[4] Banerjee S.R., Pomper M.G. (2013). Clinical applications of Gallium-68. Appl. Radiat. Isot..

[5] Decristoforo C., Knopp R., von Guggenberg E., Rupprich M., Dreger T., Hess A., Virgolini I., Haubner R. (2007). A fully automated synthesis for the preparation of 68Ga-labelled peptides. Nucl. Med. Commun..

[6] Peitl P.K., Rangger C., Garnuszek P., Mikolajczak R., Hubalewska-Dydejczyk A., Maina T., Erba P., Decristoforo C. (2019). Clinical translation of theranostic radiopharmaceuticals: Current regulatory status and recent examples. J. Label. Compd. Radiopharm..

[7] Decristoforo C., Penuelas I., Patt M., Todde S. (2017). European regulations for the introduction of novel radiopharmaceuticals in the clinical setting. Q. J. Nucl. Med. Mol. Imaging.

[8] ICH Guideline M3(R2) on Non-Clinical Safety Studies for the Conduct of Human Clinical Trials and Marketing Authorisation for Pharmaceuticals. http://www.ema.europa.eu/docs/en_GB/document_library/Scientific_guideline/2009/2009/WC500002720.pdf.

[9] Todde S., Windhorst A.D., Behe M., Bormans G., Decristoforo C., Faivre-Chauvet A., Ferrari V., Gee A.D., Gulyas B., Halldin C. (2014). EANM guideline for the preparation of an Investigational Medicinal Product Dossier (IMPD). Eur. J. Nucl. Med. Mol. Imaging.

[10] Reubi J.C., Schaer J.C., Waser B. (1997). Cholecystokinin(CCK)-A and CCK-B/gastrin receptors in human tumors. Cancer Res..

[11] Reubi J.C. (2007). Targeting CCK receptors in human cancers. Curr. Top. Med. Chem..

[12] Sanchez C., Escrieut C., Clerc P., Gigoux V., Waser B., Reubi J.C., Fourmy D. (2012). Characterization of a novel five-transmembrane domain cholecystokinin-2 receptor splice variant identified in human tumors. Mol. Cell. Endocrinol..

[13] Reubi J.C. (2003). Peptide receptors as molecular targets for cancer diagnosis and therapy. Endocr. Rev..

[14] Klingler M., Summer D., Rangger C., Haubner R., Foster J., Sosabowski J., Decristoforo C., Virgolini I., von Guggenberg E. (2019). DOTA-MGS5, a New Cholecystokinin-2 Receptor-Targeting Peptide Analog with an Optimized Targeting Profile for Theranostic Use. J. Nucl. Med. Off. Publ. Soc. Nucl. Med..

[15] Roosenburg S., Laverman P., Joosten L., Eek A., Rutjes F.P.J.T., van Delft F.L., Boerman O.C. (2012). In Vitro and In Vivo Characterization of Three 68Ga- and 111In-Labeled Peptides for Cholecystokinin Receptor Imaging. Mol. Imaging.

[16] Klingler M., Hormann A.A., Guggenberg E.V. (2021). Cholecystokinin-2 receptor targeting with radiolabeled peptides: Current status and future directions. Curr. Med. Chem..

[17] Sauter A.W., Mansi R., Hassiepen U., Muller L., Panigada T., Wiehr S., Wild A.M., Geistlich S., Behe M., Rottenburger C. (2019). Targeting of the Cholecystokinin-2 Receptor with the Minigastrin Analog (177)Lu-DOTA-PP-F11N: Does the Use of Protease Inhibitors Further Improve In Vivo Distribution?. J. Nucl. Med. Off. Publ. Soc. Nucl. Med..

[18] Klingler M., Decristoforo C., Rangger C., Summer D., Foster J., Sosabowski J.K., von Guggenberg E. (2018). Site-specific stabilization of minigastrin analogs against enzymatic degradation for enhanced cholecystokinin-2 receptor targeting. Theranostics.

[19] Laverman P., Joosten L., Eek A., Roosenburg S., Peitl P.K., Maina T., Mäcke H., Aloj L., Von Guggenber E., Sosabowski J.K. (2011). Comparative biodistribution of 12 111In-labelled gastrin/CCK2 receptor-targeting peptides. Eur. J. Nucl. Med. Mol. Imaging.

[20] Rottenburger C., Nicolas G.P., McDougall L., Kaul F., Cachovan M., Vija A.H., Schibli R., Geistlich S., Schumann A., Rau T. (2020). Cholecystokinin 2 Receptor Agonist 177Lu-PP-F11N for Radionuclide Therapy of Medullary Thyroid Carcinoma: Results of the Lumed Phase 0a Study. J. Nucl. Med..

[21] Hubalewska-Dydejczyk A., Mikolajczak R., Decristoforo C., Kolenc-Peitl P., Erba P.A., Zaletel K., Maecke H., Maina T., Konijnenberg M., Garnuszek P. (2019). Phase I clinical trial using a novel CCK2 receptor-localizing radiolabelled peptide probe for personalized diagnosis and therapy of patients with progressive or metastatic medullary thyroid carcinoma—final results. Eur. J. Nucl. Med. Mol. Imaging.

[22] Erba P.A., Maecke H., Mikolajczak R., Decristoforo C., Zaletel K., Maina-Nock T., Peitl P.K., Garnuszek P., Froberg A., Goebel G. (2018). A novel CCK2/gastrin receptor-localizing radiolabeled peptide probe for personalized diagnosis and therapy of patients with progressive or metastatic medullary thyroid carcinoma: A multicenter phase I GRAN-T-MTC study. Pol. Arch. Intern. Med. Pol. Arch. Med. Wewn..

[23] Grzmil M., Qin Y., Schleuniger C., Frank S., Imobersteg S., Blanc A., Spillmann M., Berger P., Schibli R., Behe M. (2020). Pharmacological inhibition of mTORC1 increases CCKBR-specific tumor uptake of radiolabeled minigastrin analogue [Lu-177]Lu-PP-F11N. Theranostics.

[24] Kolenc Peitl P., Tamma M., Kroselj M., Braun F., Waser B., Reubi J.C., Sollner Dolenc M., Maecke H.R., Mansi R. (2015). Stereochemistry of Amino Acid Spacers Determines the Pharmacokinetics of ^111^In-DOTA-Minigastrin Analogues for Targeting the CCK2/Gastrin Receptor. Bioconjug. Chem..

[25] Maina T., Nock B.A., Zhang H., Nikolopoulou A., Waser B., Reubi J.C., Maecke H.R. (2005). Species differences of bombesin analog interactions with GRP-R define the choice of animal models in the development of GRP-R-targeting drugs. J. Nucl. Med. Off. Publ. Soc. Nucl. Med..

[26] Hofsli E., Thommesen L., Norsett K., Falkmer S., Syversen U., Sandvik A., Laegreid A. (2002). Expression of chromogranin A and somatostatin receptors in pancreatic AR42J cells. Mol. Cell. Endocrinol..

[27] Caplin M.E., Clarke P., Grimes S., Dhillon A.P., Khan K., Savage K., Lewin J., Michaeli D., Pounder R.E., Watson S.A. (1999). Demonstration of new sites of expression of the CCK-B/gastrin receptor in pancreatic acinar AR42J cells using immunoelectron microscopy. Regul. Pept..

[28] Smith T., Petoussi-Henss N., Zankl M. (2000). Comparison of internal radiation doses estimated by MIRD and voxel techniques for a “family” of phantoms. Eur. J. Nucl. Med..

[29] Optimizing Oncologic FDG-PET/CT Scans to Decrease Radiation Exposure. https://www.imagewisely.org/Imaging-Modalities/Nuclear-Medicine/Optimizing-Oncologic-FDG-PETCT-Scans.

[30] Radiological Protection in Biomedical Research. https://www.icrp.org/publication.asp?id=ICRP%20Publication%2062.

[31] Virgolini I., Ambrosini V., Bomanji J.B., Baum R.P., Fanti S., Gabriel M., Papathanasiou N.D., Pepe G., Oyen W., De Cristoforo C. (2010). Procedure guidelines for PET/CT tumour imaging with Ga-68-DOTA-conjugated peptides: Ga-68-DOTA-TOC, Ga-68-DOTA-NOC, Ga-68-DOTA-TATE. Eur. J. Nucl. Med. Mol. Imaging.

[32] Sandstrom M., Velikyan I., Garske-Roman U., Sorensen J., Eriksson B., Granberg D., Lundqvist H., Sundin A., Lubberink M. (2013). Comparative biodistribution and radiation dosimetry of 68Ga-DOTATOC and 68Ga-DOTATATE in patients with neuroendocrine tumors. J. Nucl. Med. Off. Publ. Soc. Nucl. Med..

[33] Pfob C.H., Ziegler S., Graner F.P., Kohner M., Schachoff S., Blechert B., Wester H.J., Scheidhauer K., Schwaiger M., Maurer T. (2016). Biodistribution and radiation dosimetry of (68)Ga-PSMA HBED CC-a PSMA specific probe for PET imaging of prostate cancer. Eur. J. Nucl. Med. Mol. Imaging.

[34] Konijnenberg M.W., Breeman W.A.P., de Blois E., Chan H.S., Boerman O.C., Laverman P., Kolenc-Peitl P., Melis M., de Jong M. (2014). Therapeutic application of CCK2R-targeting PP-F11: Influence of particle range, activity and peptide amount. EJNMMI Res..

[35] Oberdiac P., Mineur L. (2010). Normal tissue tolerance to external beam radiation therapy: The stomach. Cancer Radiother..

[36] Aloj L., Caraco C., Panico M., Zannetti A., Del Vecchio S., Tesauro D., De Luca S., Arra C., Pedone C., Morelli G. (2004). In vitro and in vivo evaluation of In-111-DTPAGlu-G-CCK8 for cholecystokinin-B receptor imaging. J. Nucl. Med..

[37] Hormann A.A., Klingler M., Rezaeianpour M., Hormann N., Gust R., Shahhosseini S., Guggenberg E.V. (2020). Initial In Vitro and In Vivo Evaluation of a Novel CCK2R Targeting Peptide Analog Labeled with Lutetium-177. Molecules.

[38] Ellis R.E. (1961). The distribution of active bone marrow in the adult. Phys. Med. Biol..

[39] Kolenc-Peitl P., Mansi R., Tamma M., Gmeiner-Stopar T., Sollner-Dolenc M., Waser B., Baum R.P., Reubi J.C., Maecke H.R. (2011). Highly improved metabolic stability and pharmacokinetics of indium-111-DOTA-gastrin conjugates for targeting of the gastrin receptor. J. Med. Chem..

[40] Kunikowska J., Ziemnicka K., Pawlak D., Ruchala M., Kolasa A., Janicka-Jedynska M., Wozniak A., Mikolajczak R., Krolicki L. (2016). Medullary thyroid carcinoma*—*PET/CT imaging with 68Ga-labelled gastrin and somatostatin analogues. Endokrynol. Pol..

